# Network analysis of chronic disease among middle-aged and older adults in China: a nationwide survey

**DOI:** 10.3389/fpubh.2025.1551034

**Published:** 2025-04-09

**Authors:** Chen Chen, Hongfeng Wu, Likun Yang, Ke Kan, Xinping Zhang, Su Zhang, Rufu Jia, Xian Li

**Affiliations:** ^1^School of Nursing, Chengde Medical University, Chengde, China; ^2^Department of Medical Development, Hebei General Hospital, Shijiazhuang, China; ^3^Supply Department, Hebei General Hospital, Shijiazhuang, China; ^4^Wound and Stomy Clinic, Hebei General Hospital, Shijiazhuang, China; ^5^School of Nursing, Hebei University of Chinese Medicine, Shijiazhuang, China; ^6^Department of President, Hebei Children’s Hospital, Shijiazhuang, China; ^7^Department of Nursing, Peking University People’s Hospital, Beijing, China; ^8^Department of Nursing, Cangzhou Central Hospital, Cangzhou, China

**Keywords:** multimorbidity, network analysis, random forest model, influencing factor, middle-aged and older people, China, CHARLS

## Abstract

**Background:**

Given the rising prevalence of chronic diseases and multimorbidity among middle-aged and older individuals in China, it is crucial to explore the patterns of chronic disease multimorbidity and uncover the underlying mechanisms driving the co-existence of multiple chronic conditions.

**Methods:**

This study analyzed data from 19,206 participants in the China Health and Retirement Longitudinal Study (CHARLS 2018). The IsingFit model was used to build the chronic disease co-morbidity network, where nodes represented diseases and edges reflected conditionally independent partial correlations. Community detection identified groups of closely related diseases using the Louvain algorithm. Multivariable linear regression with forward stepwise selection explored factors influencing chronic disease co-morbidity. A random forest model ranked these factors by importance, providing insights into relationships and key contributors.

**Results:**

This study identified the most frequent multimorbidity pairs in the middle-aged and older adult population as hypertension with arthritis, and digestive diseases with arthritis. Multimorbidities were classified into four subgroups: respiratory diseases, metabolic syndrome, neurological diseases, and digestive diseases. Heart disease showed centrality in the multimorbidity network, while memory-related diseases played a bridging role. Key factors associated with multimorbidity included age, gender, pain, sleep, physical activity, depression, and education. Random forest analysis revealed that age and pain had the greatest impact on multimorbidity development, offering insights for targeted prevention and management strategies.

**Conclusion:**

This study systematically analyzed multimorbidity patterns and their influencing factors in the Chinese middle-aged and older adult population. The data were examined at three levels: overall network, key influencing factors, and individual characteristics. Cardio-metabolic diseases were identified as a core component of the multimorbidity network. Advanced age, pain, and depression were found to be independent risk factors affecting the number of multimorbidities, while healthy behaviors acted as significant protective factors. The study enhances understanding of multimorbidity mechanisms and provides a scientific basis for public health interventions, emphasizing the importance of behavioral modification, health education, and social support for high-risk groups.

## 1 Introduction

In the society of rapid aging, the increasing prevalence of chronic diseases among middle-aged and older adults poses a key public health challenge. By 2040, 28% of China’s population will be aged 60 years and older, numbering 402 million (1, 2), and it is expected that more than 75% of this population will suffer from at least one chronic disease (3). Globally, approximately one-third of individuals with chronic diseases develop multimorbidity (4), defined as the presence of two or more chronic conditions, with the risk significantly increasing with age. Compared to those with a single chronic condition, individuals with multimorbidity face a higher likelihood of premature mortality, prolonged hospital admissions, and extended hospital stays, placing substantial burdens on patients, society, and healthcare systems (5, 6). These burdens include elevated healthcare costs, increased complexity in clinical management, and challenges related to medication adherence (7). Despite multimorbidity’s rising prevalence and severity, effective treatment approaches remain limited (8). The current gaps in understanding the patterns and determinants of chronic disease multimorbidity impede the development of effective prevention and management frameworks (9). Strengthening research on multimorbidity is therefore essential.

Network analysis, an emerging statistical method, has been increasingly utilized in recent years to study complex systems, including social networks (10) and biological networks (11, 12). Its application to multimorbidity networks has attracted increasing attention from researcher. As exemplified by Chen et al., disease trajectory network analysis was employed to visualize comorbidity relationships using longitudinal datas (13). By constructing multimorbidity networks, chronic diseases can be represented as nodes in the network, while comorbidity relationships between diseases are depicted as edges connecting these nodes (14). The co-occurrence frequency of different diseases can be represented by the weights of the edges, while node-specific metrics such as degree and centrality can be used to evaluate the importance of individual diseases within the network (15). It is noteworthy that few studies have constructed co-morbidity networks using large sample sizes. Most existing studies have only visualized these networks without statistical validation (16). The relationships between diseases and their roles in the networks remain inadequately validated and explained. This limitation has hindered the identification of comorbidities as targets for interventions and the development of public health policies.

This study aims to address the research gap by analyzing data from the China Health and Retirement Longitudinal Study (CHARLS) to explore multimorbidity in the Chinese population aged 45 years and older. Its primary objective is to characterize the comorbidity network of chronic diseases in middle-aged and older individuals in China. By applying network analysis methods, the study seeks to uncover potential disease associations that may remain undetected using traditional statistical approaches. The findings are expected to provide a new perspective for comprehensive chronic disease management, deepen understanding of multimorbidity networks in this population, and shed light on the mechanisms or underlying causes of multimorbidity. Furthermore, the study incorporates the random forest method to examine the influence of demographic and lifestyle factors on multimorbidity, thereby building a robust scientific foundation for the development of targeted intervention strategies.

## 2 Methods

### 2.1 Background of the study

We used data from the 2018 CHARLS (China Health and Retirement Longitudinal Study), a nationally representative longitudinal survey of Chinese individuals aged ≥45 years and their spouses, approved by the Biomedical Ethics Committee of Peking University (IRB00001052-11015). CHARLS is an interdisciplinary research project aimed at representing Chinese residents aged 45 years and older, with no upper age limit. The survey used a stratified sampling strategy, selecting participants from urban and rural counties based on per capita GDP. A multistage random sampling process was implemented, starting at the county or district level, followed by the village or community level, and finally selecting households. The baseline survey, conducted in 2011–2012, included 17,708 participants from 150 counties. Subsequent follow-up surveys were carried out in 2013 (Wave 2), 2015 (Wave 3), and 2018 (Wave 4). To ensure sample representativeness, the baseline survey covered 150 counties/districts and 450 villages/urban communities nationwide, involving 17,708 individuals from 10,257 households. By 2020, five waves of data collection had been completed (2011, 2013, 2015, 2018, and 2020). Ethical approval for all the CHARLS waves was granted from the Institutional Review Board at Peking University. Detailed descriptions of the CHARLS design and data collection procedures are available in previous publications and on the official website (17, 18).

### 2.2 Study population

**Figure 1:**
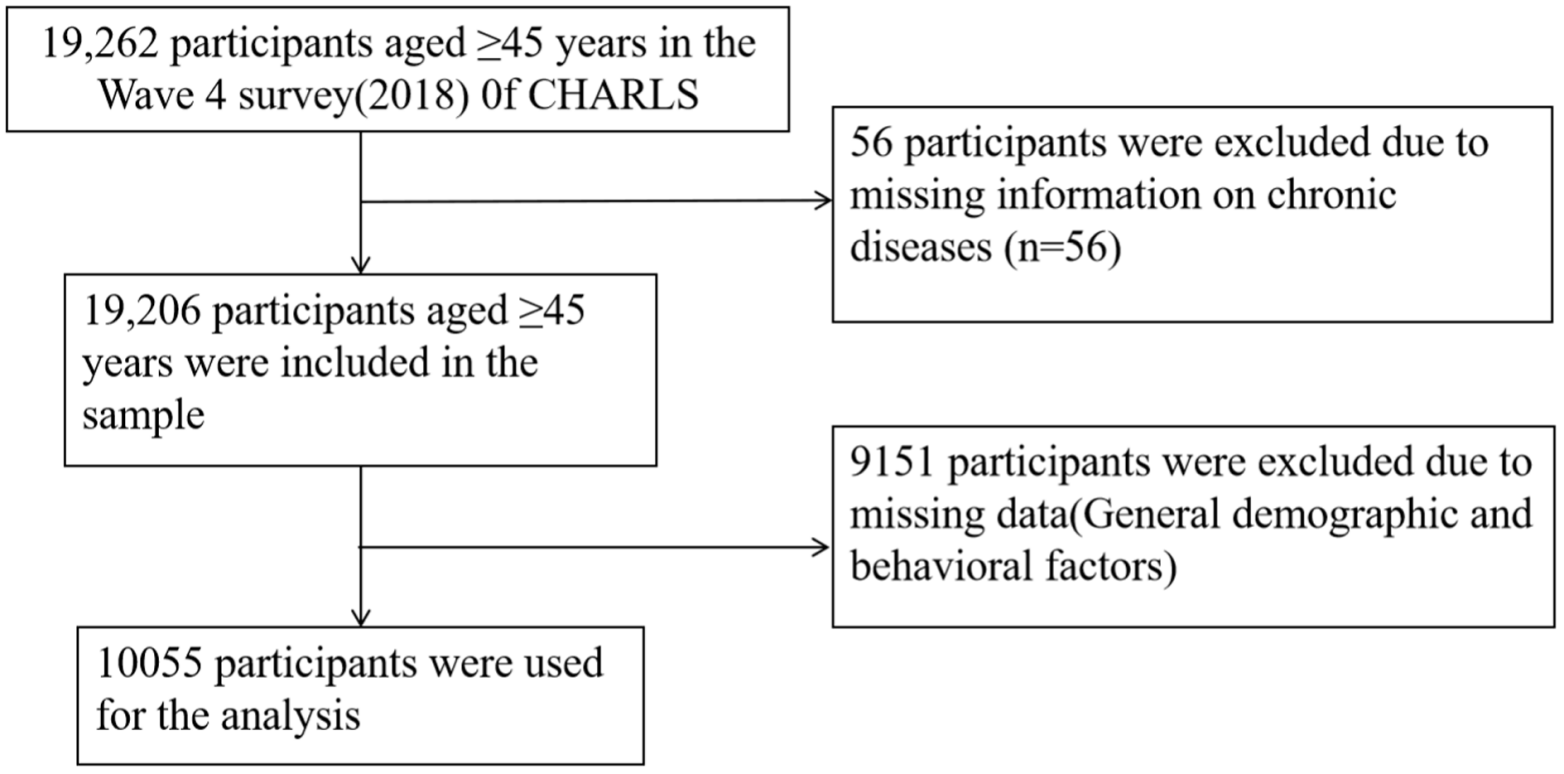
Sample flowchart.

This study utilized data from the 2018 wave of the China Health and Retirement Longitudinal Study (CHARLS). The primary focus was on individuals aged 45 years and older. In 2018, 19,206 participants were screened (17). After excluding participants with missing covariate data, the final analysis included 10,055 individuals (Figure 1).

### 2.3 Diseases selection

The chronic diseases included in this study were hypertension (HTN), cancer, diabetes (DM), heart disease (HD), chronic lung disease (CLD), stroke (STK), memory-related diseases (MRD), arthritis (AR), dyslipidemia (DLP), liver disease (LD), kidney disease (KD), digestive diseases (DD), and asthma (AST), all of which were self-reported by the participants. The diagnosis and onset time of 14 common chronic diseases were collected in baseline and every follow-up through questionnaire. Participants were first asked, “Have you been diagnosed with …(disease) by a doctor?.” If the answer was yes, a value of 1 was assigned; if the answer was no, a value of 0 was assigned. The final count of chronic diseases among adults was obtained by summing up these values based on the participants’ responses.

### 2.4 Physical activity

The China Health and Retirement Longitudinal Study (CHARLS) collected data on the number of days participants engaged in at least 10 min of physical activity per day during the past week, as well as the duration of these activities each day. Physical activity levels were evaluated using the method proposed by Li et al. (19), which assigns metabolic equivalents (METs) to different intensities of physical activity: 3.3 for low-intensity activities, 4.0 for moderate-intensity activities, and 8.0 for high-intensity activities. Weekly energy expenditure was calculated using the formula: $\begin{array}{l} \mathrm{Weekly energy expenditure}=\mathrm{MET}\times \mathrm{daily activity duration}\;\\ \left(\mathrm{minutes}\right)\times \mathrm{days of activity}\;\\ \mathrm{per}\;\mathrm{week}\;\left(\mathrm{days}\right)\text{.} \end{array}$ Physical activity levels were categorized based on the International Physical Activity Questionnaire (IPAQ) criteria into three groups: low physical activity (<600 MET-minutes/week), moderate physical activity (600 ~ 3,000 MET-minutes/week), and high physical activity (>3,000 MET-minutes/week) (20, 21).

### 2.5 Sleep

Sleep quality was assessed using a self-reported question asking participants about their average nightly sleep duration over the past month. Participants provided their responses in hours and minutes. Based on the participants’ answers, the sleep duration was divided into three categories: short sleep duration (≤ 6 h), medium sleep duration (6–8 h), and long sleep duration (> 8 h) (22).

### 2.6 Cognitive function

Cognitive function was assessed using the Chinese version of the Mini-Mental State Examination (MMSE), which consists of 24 items across four cognitive domains (23). The total score ranges from 0 to 24, with lower scores indicating poorer cognitive function (24).

### 2.7 Depression

Depression was assessed using the Chinese version of the Center for Epidemiological Survey Depression Scale (CES-D-10). The CES-D-10 employs a 4-point Likert scale with the following response options: “None,” “Rarely (1–2 days),” “Sometimes (3–4 days),” and “Always (5–7 days).” Two items were reverse-scored, while the remaining items were scored positively, with individual item scores ranging from 0 to 3. The total score ranges from 0 to 30, with higher scores indicating more severe depressive symptoms. A score of ≥10 was used to identify depression (25, 26). The CES-D-10 has been fully validated in the Chinese older adult population to demonstrate its satisfactory reliability and validity (27).

### 2.8 Disability

Disability was assessed using the Physical Self-Maintenance Scale (PSMS) to evaluate activities of daily living (ADL). The scale consists of six components. In this study, participants were classified as disabled if they responded “I have difficulty and need help” or “I am unable to do it” to any of the items (28).

### 2.9 Covariates

The study included covariates such as sociodemographic factors, health behaviors, and sensory function. Sociodemographic factors encompassed age, education level (classified into four categories: illiterate, primary school, middle school, and high school or above), and marital status (married or unmarried). Health behaviors included smoking status (Yes/No) and alcohol consumption (Yes/No). The pain is assessed by the question, “Which parts of the body are experiencing pain? Please list all affected areas.” If the participant reports pain in any body part, a value of 1 is assigned; otherwise, a value of 0 is assigned. Vision was assessed by asking participants about their regular use of corrective lenses and their ability to recognize distant objects, such as identifying a friend across the street, with glasses if applicable. Similarly, hearing was evaluated through questions about participants’ overall hearing status and the impact of hearing aids, if used. Participants rated their vision and hearing on a scale ranging from “excellent” to “poor.” Vision was categorized as “good” for ratings of “excellent,” “very good,” or “good”; “moderate” for “fair”; and “poor” for “poor.” The same classification criteria were applied to hearing.

## 3 Statistical analysis

### 3.1 Descriptive statistics

Descriptive statistics and univariate analyses were conducted using IBM SPSS (version 22.0). Continuous variables were reported as mean ± standard deviation (Mean ± SD), and categorical variables were presented as frequencies and percentages. The chi-square test was used to assess differences in categorical variables between groups, while t test was employed for continuous variables. For participants with two or more chronic conditions, each unique combination of coexisting conditions was counted as a comorbidity pair. For instance, if an individual had hypertension, dyslipidemia, and heart disease, the comorbidity pairs “hypertension & dyslipidemia,” “hypertension & heart disease,” and “dyslipidemia & heart disease” were counted separately. The prevalence of each chronic disease comorbidity pair was calculated as the ratio of individuals with a specific pair to the total sample size.

### 3.2 Network construction

The IsingFit model was used to analyze the relationships between chronic diseases. The model, designed for binary variables, uses conditionally independent partial correlations to represent relationships. In the network, nodes represent chronic diseases, and edges indicate the strength of associations between them, with edge weights reflecting the magnitude of these correlations. High-weight edges were displayed in blue, and low-weight edges were shown in light gray. The Fruchterman-Reingold algorithm was used to position highly connected or strongly associated nodes at the center of the network, while less connected nodes were placed at the periphery. To further explore the community characteristics of chronic disease comorbidity, the Louvain algorithm (resolution = 1.0) was used for community detection. The results of the community detection were distinguished by color, with each community assigned a unique color, using the “Set3” palette from the RColorBrewer package in R (version 4.4.2). The Louvain algorithm is suitable for detecting subgroups in large-scale networks and is based on modularity maximization. By analyzing the community partition results, we can identify the grouping patterns of different diseases in the chronic disease comorbidity network. However, it also has limitations, such as the tendency to converge to local optima, especially when applied to more complex networks. Network visualization, performed using the “qgraph” package in “R” (version 4.4.2), highlighted community structures and the strength of associations, providing valuable insights into clustering patterns and potential relationships between chronic diseases.

### 3.3 Identifying central symptoms and network stability

To identify the most critical symptoms within the comorbidity network, three centrality metrics were evaluated: strength, betweenness, and closeness. Strength, the primary centrality metric in this study, was defined as the absolute sum of edge weights connected to a node, reflecting the overall importance of a disease. Nodes with higher strength scores exerted greater influence within the network. Closeness was calculated as the inverse of the total distance from a node to all other nodes, while betweenness measured the frequency with which a node appeared on the shortest paths between other nodes. Analyses were conducted using the “networktools” and “qgraph” packages in R (version 4.4.2). Centrality metrics were standardized as z-scores to facilitate comparisons between nodes. Network stability was assessed using the bootnet function, which performed 1,000 bootstrap samples to compute stability indices for edge weights, strength, closeness, and betweenness. The subset bootstrap method was used to calculate the correlation stability coefficient (CS-C) for centrality metrics, with a CS-C value of ≥0.25, and ideally >0.5, considered indicative of acceptable stability.

### 3.4 Analysis of influencing factors

Univariate analysis to select predictors of multimorbidities was conducted using Lasso regression with the “glment” package in R (version 4.4.2). Lasso regression, based on ordinary least squares, penalizes the absolute values of model coefficients using an L1 regularization term, forcing some coefficients to zero and achieving feature selection. The model was constructed using a random seed value of 317 and 10-fold cross-validation to determine the optimal regularization parameter (*λ*). This approach ensured the model’s generalization ability and helped identify key predictors with a significant impact on chronic disease comorbidities. Finally, the selected predictors were included in linear regression analysis to identify significant factors. Multivariate linear regression with forward stepwise selection was used to identify potential influences on comorbidities. Variables were selected and excluded based on thresholds set at αin = 0.05 and αout = 0.10, respectively. The statistical significance level was set at *p* < 0.05, and a random forest model was built using the Random Forest package in R (version 4.4.2). The Random Forest package has strong nonlinear modeling capabilities and is more tolerant to missing data, improving model accuracy by constructing multiple decision trees and combining their predictions. Each tree is trained on a randomly selected subset of samples and features, reducing the risk of overfitting. The model was seeded with a value of 42, using 500 trees, and the relative importance of each predictor was assessed through ranked importance analysis. Ten-fold cross-validation was used to verify the model’s fit. Furthermore, the Network Comparison Test function in R (version 4.4.2) was employed to perform network comparisons, thereby elucidating the network disparities in comorbidities across diverse populations.

## 4 Results

### 4.1 Participant characteristics

The study included 19,206 middle-aged and older adults (aged 45 years and above) from China for comorbidity network analysis. After excluding participants with missing covariate data, 10,055 individuals remained in the final analysis, including 5,550 males (55.20%) and 4,505 females (44.81%). Of these, 5,023 participants (49.96%) were aged 45–59 years, while 5,032 (50.04%) were aged 60 years or older. Regarding educational attainment, 643 participants (6.39%) were illiterate, 4,432 (44.08%) had completed primary school, 3,045 (30.28%) had completed middle school, and 1,935 (19.24%) had attained at least a high school education. Regarding marital status, 1,036 participants (10.30%) were unmarried or not currently married, while 9,019 (89.70%) were married. Except for smoking, all between-group differences were statistically significant. The Participant characteristics were presented in Table 1.

**Table 1:** Demographic characteristics and prevalence of multimorbidity among middle-aged and older adults in China.

Variables	Categories	Overall (*n* = 10,055)	Cases (*n*, %)	*χ*^2^/*t*	*p* value
		Mean ± SD			
Cognitive function		13.25 ± 3.45	13.03 ± 3.44	6.89	<0.001
Age	<60	5,023	2,260 (44.99)	276.42	<0.001
	≥60	5,032	3,259 (64.77)		
Gender	Male	5,550	2,958 (53.30)	12.61	<0.001
	Female	4,505	2,561 (56.84)		
Education	Illiterate	643	396 (61.59)	34.61	<0.001
	Primary	4,432	2,523 (56.92)		
	Middle school	3,045	1,572 (51.62)		
	High school+	1935	1,028 (53.13)		
Marital	Married	9,019	4,862 (53.90)	33.93	<0.001
	Unmarried	1,036	657 (63.42)		
Residence	Urban	3,507	2002 (57.08)	10.51	0.001
	Rural	6,548	3,517 (53.71)		
Smoking	Yes	4,766	2,636 (55.31)	0.646	0.422
	No	5,289	2,883 (54.51)		
Drinking	Yes	3,146	1,558 (49.52)	53.22	<0.001
	No	6,909	3,961 (57.33)		
Depression	Yes	3,171	2,151 (67.83)	313.47	<0.001
	No	6,884	3,368 (48.93)		
Sleep	≤6	5,438	3,290 (60.50)	151.91	<0.001
	6 ~ 8	3,959	1898 (47.94)		
	>8	658	331 (50.30)		
Physical activity	Low	1,216	735 (5.83)	44.81	0.001
	Moderate	2,786	1,622 (58.22)		
	High	6,053	3,162 (52.0.23)		
Hearing	Fair	5,205	3,064 (58.87)	247.53	<0.001
	Good	3,870	1773 (45.81)		
	poor	980	682 (69.59)		
Vision	Fair	3,778	2,280 (60.34)	193.87	<0.001
	Good	4,358	2049 (47.02)		
	Poor	1919	1,190 (62.01)		
Disability	No	5,961	3,893 (65.30)	641.94	<0.001
	Yes	4,094	1,626 (39.71)		
Pain	No	4,200	1731 (41.21)	544.65	<0.001
	Yes	5,855	3,788 (64.69)		

### 4.2 Prevalence of chronic disease multimorbidity in middle-aged and older adults in China

Among 19,206 middle-aged and older adult individuals aged 45 and above in China, 10,791 cases of chronic disease multimorbidity were identified, resulting in a prevalence rate of 56.19%. The prevalence of chronic disease multimorbidity varied significantly across genders, age groups, education levels, sleep, marital statuses cognitive function and depression, with all differences found to be statistically significant (*p* < 0.01; Table 1). The 10 most common multimorbidity pairs among the 19,206 individuals were primarily two-way combinations of six prevalent chronic diseases: hypertension, diabetes, dyslipidemia, arthritis or rheumatism, digestive diseases, and heart disease. Among these, the most frequent co-morbidity pairs were hypertension and arthritis or rheumatism, and digestive diseases and arthritis or rheumatism, with prevalence rates of 15.02 and 14.84%, respectively. Detailed information on the top 10 multimorbidity pairs in the middle-aged and older adult population in China is presented in Table 2.

**Table 2:** Top 10 most common comorbidity pairs among middle-aged and older adults in China.

Rank	Comorbidity pairs	Number of cases	Prevalence (%)
1	Hypertension & Arthritis	1,510	15.02
2	Arthritis & Digestive Diseases	1,492	14.84
3	Hypertension & Dyslipidemia	1,462	14.54
4	Hypertension & Heart Diseases	1,156	11.60
5	Hypertension & Digestive Diseases	1,113	11.07
6	Heart Diseases & Arthritis	1,053	10.47
7	Arthritis & Dyslipidemia	978	9.73
8	Heart Diseases & Digestive Diseases	922	9.17
9	Chronic Lung Disease & Arthritis	874	8.69
10	Heart Diseases & Dyslipidemia	868	8.63

### 4.3 Network analysis of multimorbidity network among middle-aged and older adults in China

**Figure 2:**
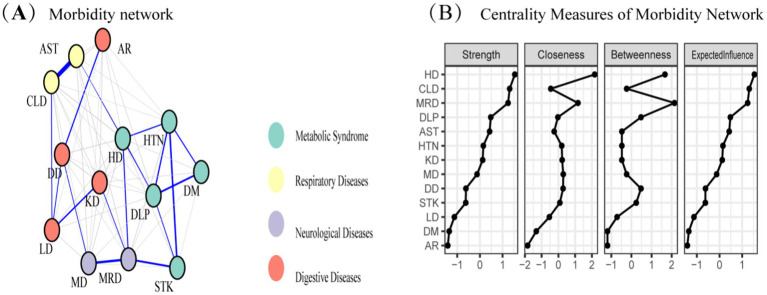
**(A)** Morbidity network showing the relationships among various diseases. Nodes represent different diseases, and edges indicate significant associations between diseases. Node colors represent disease categories: respiratory diseases (yellow), metabolic syndrome (blue), neurological diseases (purple), and digestive diseases (red). **(B)** Centrality measures of the morbidity network, including strength, closeness, betweenness, and expected influence, for each disease. These measures quantify the relative importance of each disease within the network.

**Figure 3:**
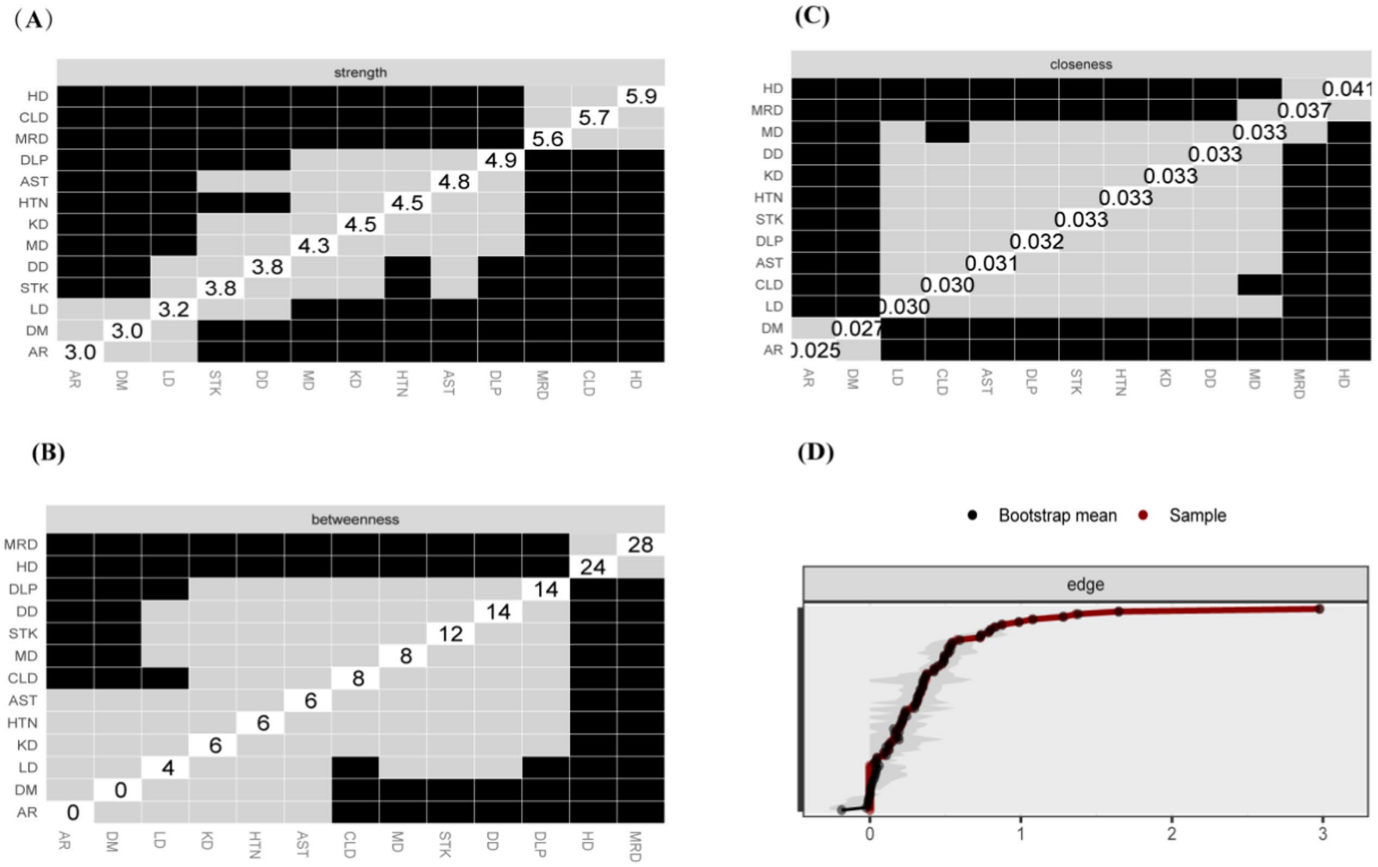
Centrality Measures and Bootstrap Validation of the Morbidity Network, **(A)** Strength centrality matrix for the morbidity network. The black cells indicate statistically significant associations between diseases based on strength centrality. **(B)** Betweenness centrality matrix for the morbidity network. The black cells represent statistically significant relationships based on betweenness centrality. **(C)** Closeness centrality matrix for the morbidity network. Statistically significant associations between diseases are highlighted in black. **(A–C)** Both the X and Y axes represent the chronic diseases included in this study. **(D)** Bootstrap validation of edge weights in the morbidity network. The red line represents the bootstrap mean, while the black dots represent the sample values. Statistically significant edges are indicated.

**Figure 4:**
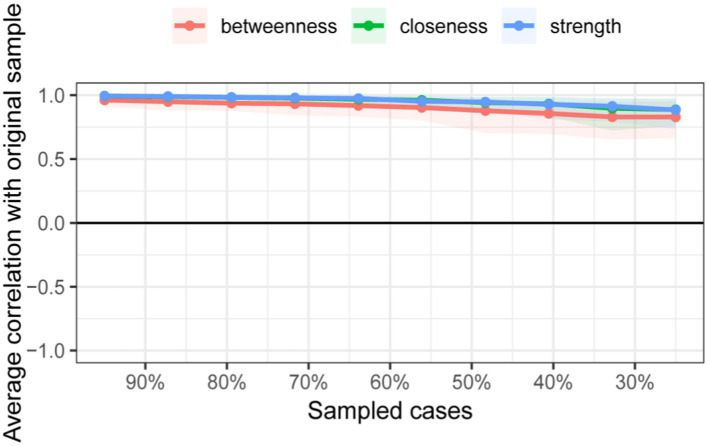
Stability of centrality measures under subsampling. The x-axis indicates the percentage of cases of the original sample included at each step. The y-axis indicates the average of correlations between the expected influence centrality index from the original network and the expected influence centrality index from the networks that were re-estimated after excluding increasing percentages of cases.

The study identified four comorbidity subgroups in the Chinese middle-aged and older adult population: metabolic syndrome, respiratory diseases, neurological diseases, and digestive diseases (Figure 2A). Due to the high independence of cancer, it was not included in the comorbidity network in this study. The strength scores of heart disease (HD), chronic lung disease (CLD), memory-related diseases (MRD), and dyslipidemia (DLP) were ranked in descending order (Figure 2B). Most differences in strength scores were statistically significant compared to other chronic diseases, indicating that these conditions are the most closely associated within the network (Figure 3A). Betweenness scores ranked memory-related diseases (MRD) and heart disease (HD) in descending order, with significant differences observed, highlighting their critical bridging roles in the disease transmission network (Figure 3B). Similarly, closeness scores ranked heart disease and memory-related diseases at the top, with statistically significant differences compared to most other diseases (Figure 3C), indicating their central roles in the network. Further bootstrap analyses confirmed that the mean edge strengths were consistent with the sample results, exhibiting narrow confidence intervals, thereby reinforcing the robustness of the analysis (Figure 3D). The central stability coefficients of the expected impacts, calculated using the sample descent bootstrap method, were all greater than 0.50, indicating sufficient stability of the expected effects (Figure 4). Additionally, the network structure remained stable even when the sample size was reduced or nodes were re-estimated, further validating the reliability and robustness of the results.

### 4.4 LASSO screening and random forest analysis of predictors for multimorbidity in middle-aged and older adults in China

**Figure 5:**
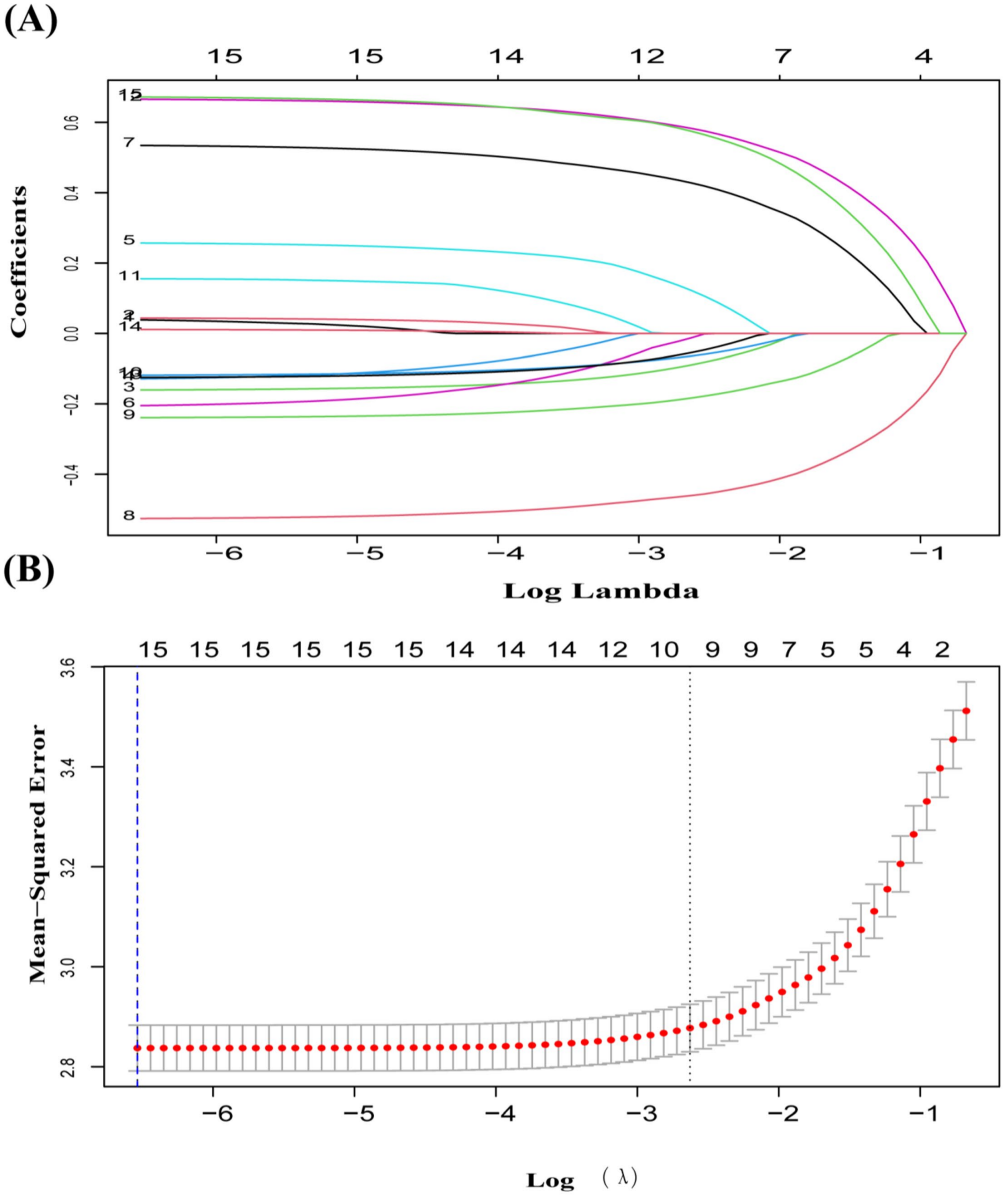
**(A)** Coefficient curves for 15 predictive factors using LASSO regression. **(B)** LASSO regression with 10-fold cross-validation was used to choose the most appropriate predictive factors. A vertical dashed line at the optimal λ value was drawn using the 1-SE criterion. At λ = 0.0014, 15 non-zero coefficients were chosen.

**Figure 6:**
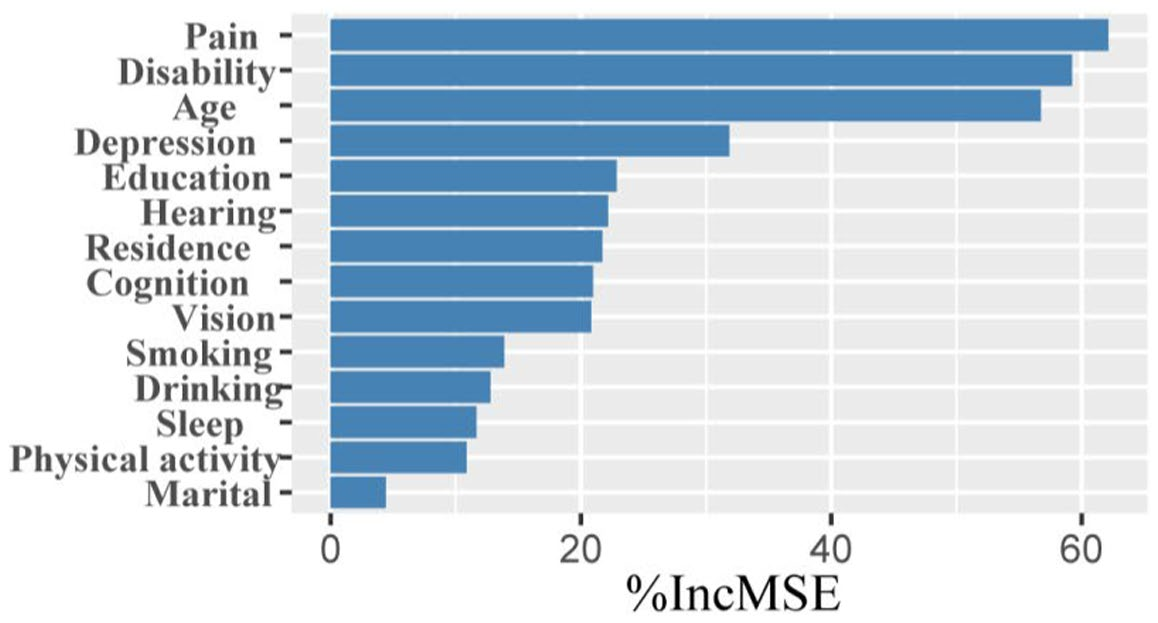
Ranking of factors in order of importance. The y-axis represents variables that were found to be associated with the number of comorbidities through multivariable linear regression analysis.

LASSO screening indicated that the model exhibited the least variation at *λ* = 0.0014, identifying 15 significant predictors with non-zero regression coefficients. It employed 10-fold cross-validation with a random seed of 317. The variable assignment method is provided in Table 3. The selected predictors included place of residence, age, gender, smoking, marital status, education, sleep, cognitive function, disability, vision, hearing, physical activity, depression, drinking, and pain (Figures 5A,B). These variables were subsequently incorporated into a multiple linear regression model, with the regression analysis results presented in Table 4. After excluding gender, a random forest approach was applied to rank the remaining variables. The results showed that pain and age were the most influential risk factors, with the full ranking results displayed in Figure 6. Notably, the 10-fold cross-validation yielded an R^2^ of 17.23%, suggesting that the model has limited explanatory power. However, it primarily serves as a non-parametric supplement. When combined with the linear regression results, it further confirms that pain and age are key factors influencing multimorbidity.

**Table 3:** Categorical variable assignment.

Categorical variable	Variable assignment
Age	<60 = 0,≥60 = 1
Gender	Female = 0, Male = 1
Education	Illiterate = 0, Primary = 1, Middle school = 2, High school+ = 3
Marital	Unmarried = 0, Married = 1
Residence	Rural = 0, Urban = 1
Smoking	Yes = 1, No = 0
Drinking	Yes = 1, No = 0
Depression	Yes = 1, No = 0
Sleep	≤6 = 0, 6 ~ 8 = 1,>8 = 2
Physical activity	Low = 0, Moderate = 1, High = 2
Hearing	Poor = 0, Fair = 1, Good = 2
Vision	Poor = 0, Fair = 1, Good = 2
Disability	Yes = 1, No = 0
Pain	Yes = 1, No = 0

**Table 4:** Results of multivariate linear regression analysis of factors associated with multimorbidity.

Variable	B	*β*	*t*	SE	95%CI	*P* value
Pain	0.665	0.175	18.015	0.037	0.593 ~ 0.737	<0.001
Age	0.677	0.181	18.512	0.037	0.605 ~ 0.749	<0.001
Disability	−0.525	−0.139	−14.135	0.037	−0.598 ~ −0.452	<0.001
Depression	0.536	0.133	13.761	0.039	0.459 ~ 0.612	<0.001
Hearing	−0.241	−0.081	−8.354	0.029	−0.298 ~ −0.184	<0.001
Residence	0.257	0.065	6.756	0.038	0.182 ~ 0.331	<0.001
Sleep	−0.161	−0.053	−5.778	0.028	−0.216 ~ −0.106	<0.001
Physical activity	−0.126	−0.047	−5.193	0.024	−0.174 ~ −0.078	<0.001
Vision	−0.119	−0.048	−5.024	0.024	−0.166 ~ −0.073	<0.001
Drinking	−0.202	−0.050	−5.106	0.040	−0.279 ~ −0.124	<0.001
Smoking	0.184	0.049	4.975	0.037	0.112 ~ 0.257	<0.001
Education	0.047	0.022	2.102	0.023	0.003 ~ 0.091	0.036
Marital	−0.130	−0.021	−2.296	0.057	−0.241 ~ −0.019	0.002
Cognitive function	0.012	0.021	2.054	0.006	0.001 ~ 0.023	0.040

### 4.5 Sensitivity analysis

We performed regression analyses separately for different gender subgroups to explore the factors influencing the number of multimorbidities in each group. The findings from these subgroup analyses are consistent with the results of our main analysis. We found that smoking worsens the number of chronic disease multimorbidities in women (Table 5), whereas alcohol consumption may act as a protective factor in men (Table 6). Other results were consistent with those from population-based studies (Table 4).

**Table 5:** Results of multivariate linear regression analysis (female).

Variable	*B*	*β*	*t*	SE	95%CI	*P* value
Pain	0.766	0.186	13.082	0.059	0.651 ~ 0.881	<0.001
Age	0.816	0.210	14.367	0.057	0.705 ~ 0.928	<0.001
Depression	0.560	0.140	9.853	0.057	0.449 ~ 0.671	<0.001
Disability	−0.467	−0.112	−7.956	0.059	−0.582 ~ −0.352	<0.001
Hearing	−0.252	−0.081	−5.726	0.044	−0.228 ~ −0.165	<0.001
Residence	0.222	0.056	3.982	0.056	0.112 ~ 0.331	<0.001
Vision	−0.160	−0.062	−4.454	0.036	−0.231 ~ −0.090	<0.001
Smoking	0.394	0.052	3.908	0.101	0.196 ~ 0.592	<0.001
Sleep	−0.164	−0.051	−3.763	0.044	−0.249 ~ −0.078	<0.001
Marital	−0.271	−0.048	−3.476	0.078	−0.423 ~ −0.107	0.001
Physical activity	−0.112	−0.040	−3.017	0.003	−0.185 ~ −0.039	0.003
Cognitive function	0.017	0.033	2.214	0.008	0.002 ~ 0.033	0.027

**Table 6:** Results of multivariate linear regression analysis (male).

Variable	*B*	*β*	*t*	SE	95%CI	*P* value
Pain	0.594	0.163	12.511	0.048	0.501 ~ 0.688	<0.001
Age	0.523	0.143	11.166	0.047	0.431 ~ 0.515	<0.001
Disability	−0.583	−0.160	−12.169	0.048	−0.677 ~ −0.489	<0.001
Depression	0.493	0.119	9.299	0.053	0.389 ~ 0.598	<0.001
Residence	0.286	0.073	5.698	0.050	0.188 ~ 0.384	<0.001
Hearing	−0.238	−0.084	−6.250	0.039	−0.313 ~ −0.164	<0.001
Drinking	−0.237	−0.065	−5.285	0.045	−0.324 ~ −0.149	<0.001
Physical activity	−0.134	−0.052	−4.190	0.032	−0.196 ~ −0.071	<0.001
Sleep	−0.149	−0.051	−4.145	0.036	−0.220 ~ −0.079	<0.001
Education	0.080	0.037	2.807	0.029	0.024 ~ 0.137	0.005
Vision	−0.087	−0.036	−2.752	0.031	−0.148 ~ −0.025	0.006

### 4.6 Comparison of networks across genders

**Figure 7:**
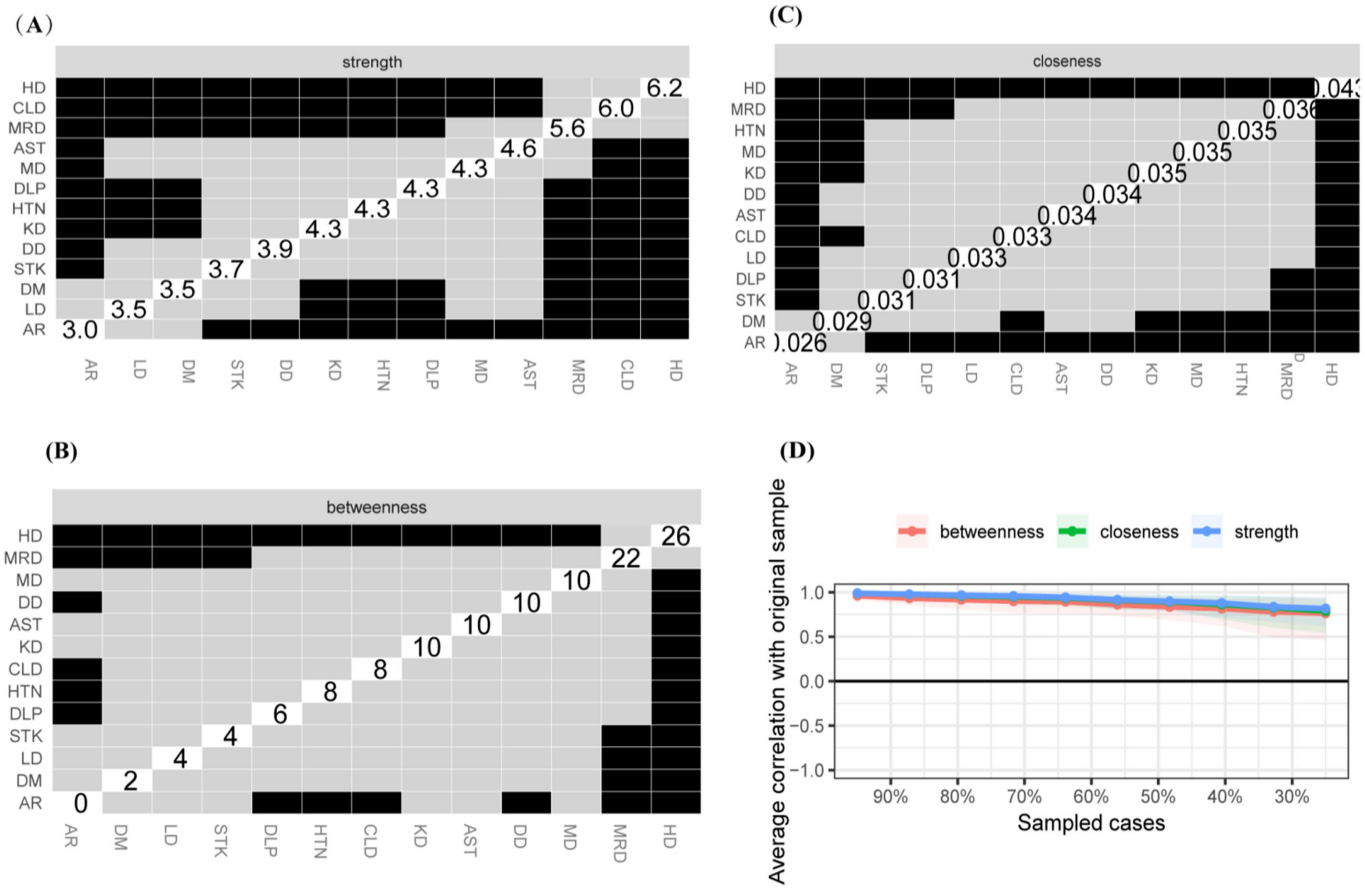
Centrality measures and bootstrap validation of the morbidity network (female). **(A)** Strength centrality matrix for the morbidity network. The black cells indicate statistically significant associations between diseases based on strength centrality. **(B)** Betweenness centrality matrix for the morbidity network. The black cells represent statistically significant relationships based on betweenness centrality. **(C)** Closeness centrality matrix for the morbidity network. Statistically significant associations between diseases are highlighted in black. **(D)** The x-axis indicates the percentage of cases of the original sample included at each step. The y-axis indicates the average of correlations between the expected influence centrality index from the original network and the expected influence centrality index from the networks that were re-estimated after excluding increasing percentages of cases.

**Figure 8:**
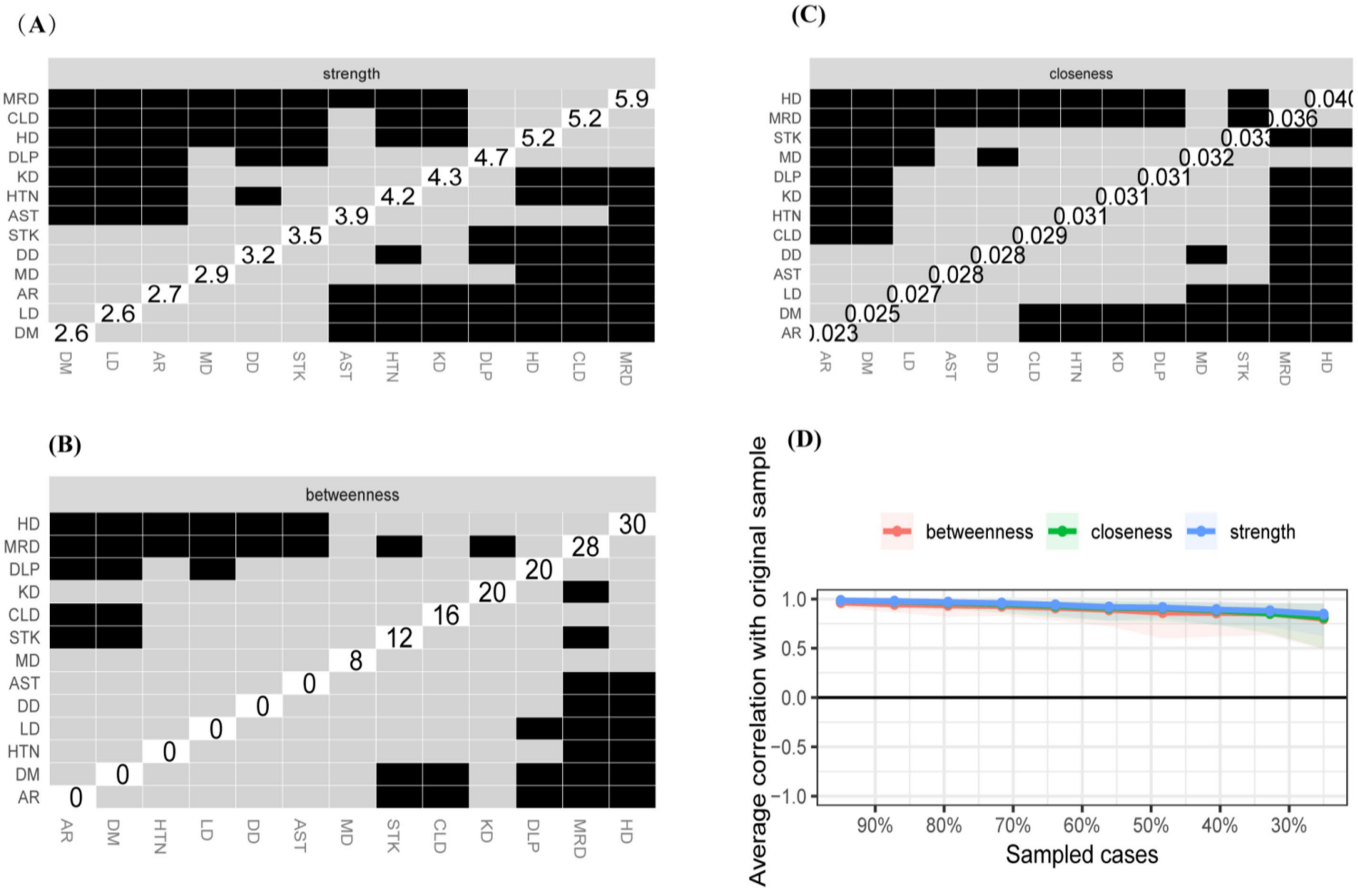
Centrality measures and bootstrap validation of the morbidity network (male). **(A)** Strength centrality matrix for the morbidity network. The black cells indicate statistically significant associations between diseases based on strength centrality. **(B)** Betweenness centrality matrix for the morbidity network. The black cells represent statistically significant relationships based on betweenness centrality. **(C)** Closeness centrality matrix for the morbidity network. Statistically significant associations between diseases are highlighted in black. **(D)** The x-axis indicates the percentage of cases of the original sample included at each step. The y-axis indicates the average of correlations between the expected influence centrality index from the original network and the expected influence centrality index from the networks that were re-estimated after excluding increasing percentages of cases.

**Figure 9:**
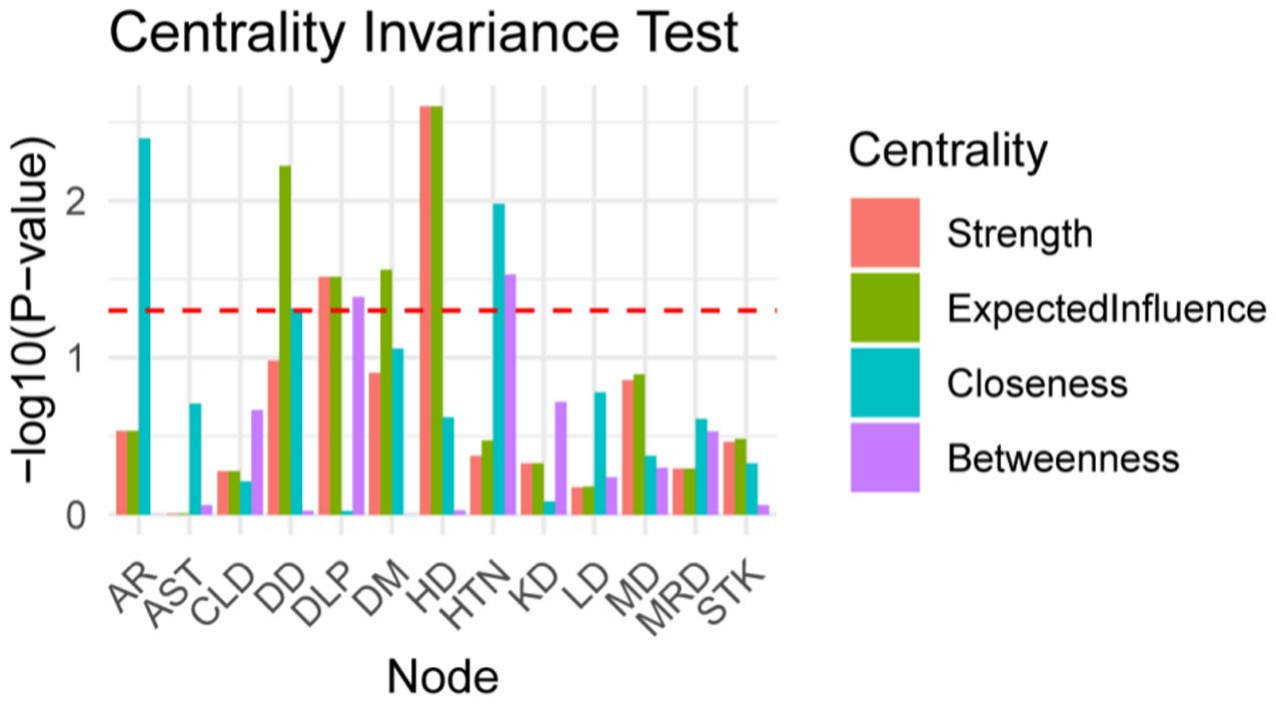
The red line represents the significance level (*P* = 0.05).

Our study examines the comorbidity networks in the original dataset from a network perspective and compares gender-based differences. In the original sample, 52.21% of participants were male. This approach minimizes bias and enhances the robustness of the findings. The findings revealed a statistically significant difference in chronic disease comorbidity networks by gender (M = 0.064, *p* = 0.045), indicating that gender influences comorbidity networks to some extent. The comorbidity network intensity was 3.726 for men and 3.858 for women, with no statistically significant difference (S = 0.132, *p* = 0.35), suggesting that there is no overall difference in network strength between the two groups. Heart disease exhibited higher values of intensity centrality, mediator centrality, and proximity centrality for both men and women, with no statistically significant difference (Figures 7–9). These results suggest that the overall network intensity is similar between genders, certain diseases, such as heart disease, remain central in both networks. The results showed that heart disease occupies a central position in the co-morbidity network of the entire population, which is consistent with findings from the whole population network. However, differences were observed at the level of individual disease nodes. In terms of intensity centrality, men exhibited the highest level of memory-related diseases, with a statistically significant difference compared to women (Figure 9). This finding suggests that memory-related diseases is more strongly associated with other diseases in male than in female.

## 5 Discussion

This study found that the prevalence of multimorbidity in China’s older population was 64.77%, consistent with the 65.6% reported by Zhang et al. based on CHARLS 2018 data (29), and significantly higher than the 41.15% reported by Cheng et al. using CHARLS 2015 data (30). This difference underscores the severity of multimorbidity in China’s aging population. In recent years, changes in lifestyle and increased life expectancy have contributed to a rising prevalence of chronic diseases among China’s middle-aged and older populations (5). This trend poses a significant health challenge and a serious strain on China’s public health system. This study suggests that hypertension, arthritis, gastric diseases, and metabolic disorders are the most common multimorbidities among middle-aged and older adult individuals in China. When considering only the prevalence of binary comorbidity groups, the frequent co-occurrence of conditions such as hypertension and arthritis aligns with the findings of Li et al. (31).

However, multimorbidity is a complex phenomenon that cannot be adequately explained by a simple superposition of diseases. Contrary to earlier research, this study unveils the intricate nature of chronic disease co-morbidities in China’s middle-aged and older adult populations from a holistic standpoint, it involves synergistic effects and complex interaction mechanisms between them (32, 33). These mechanisms involve various factors, including chronic inflammation, metabolic disorders, immune dysfunction, and psychosocial stress (34). The study indicated that chronic disease multimorbidity could be classified into four major subgroups: metabolic syndromes, respiratory diseases, neurological diseases, and digestive diseases. This classification aligns with the study by Olaya et al. (35). The existence of significant associations between diseases within or across subgroups suggests that multimorbidities affect multiple organ systems through simultaneous effects (36). Centrality analysis revealed that heart disease scored highly in intensity centrality, proximity centrality, and betweenness centrality. This suggests that heart disease occupies a central position in the network of multimorbidity and have a broad impact on other diseases. These findings are similar to the conclusions of Li and Wang (CHARLS 2018) (37) and Liu and Chen (CHARLS 2020) (38), both of which identified hypertension as the central component of the comorbidity network, simply through point size and edge weights. Both diseases are classified as cardiovascular diseases, they are associated with a range of chronic diseases that share independent risk factors, including stroke, diabetes, and kidney disease. In the future, the prevention and management of multimorbidities can be achieved by addressing common risk factors and developing preventive and treatment guidelines with a focus on heart disease and hypertension. This approach has the potential to effectively reduce the incidence of chronic disease multimorbidities in older adult individuals or decelerate their progression. In addition, memory-related disorders were found to play a significant bridging role in the comorbidity network. These disorders were closely linked to the high prevalence of cognitive dysfunction in the older adult population and its bidirectional relationship with other chronic diseases, the reasons for this can be analyzed as follows. Cognitive dysfunction exacerbates the onset and progression of other chronic diseases by impairing patients’ ability to self-manage (39). In the future, this approach could serve as an mediating Target to reduce the prevalence of multimorbidity. The results of the bootstrap test showed that the stability of edge strength and node centrality was high, indicating that the network analysis results were highly robust and further confirming the strong association between the complex interactions of diseases in this study and the risk of multimorbidity.

In this study, LASSO regression was used to identify 15 significant predictors. Random forest analysis assessed the significance of the variables using non-parametric methods, particularly in the presence of potential non-linear relationships, providing valuable quantitative insights for targeted interventions. In random forest analysis, age emerged as the primary factor, emphasizing its significance in multimorbidity risk. This finding is consistent with the conclusion of Song et al. (40) and underscores the importance of successful aging. Disability is another significant factor influencing the number of multimorbidities. However, the present study found a negative association between incapacitation and the number of multimorbidities, suggesting that incapacitation may serve as a protective factor. This contradicts the findings of Xu et al. (41), who used the incidence of multimorbidities as an outcome indicator. Given the limitations in the definition of incapacitation in this study, further exploration is needed to clarify the relationship between multimorbidity and incapacitation. Chronic pain, as an independent risk factor for chronic disease comorbidity, is not only a common symptom across various chronic diseases but also has the potential to worsen other chronic conditions through several mechanisms (42). These mechanisms include increasing psychological stress, triggering sleep disturbances (43), and limiting daily activities, all of which indirectly contribute to the worsening of other chronic diseases (44). This can accelerate the onset and progression of multiple chronic conditions. Pain management has become a critical component in the prevention and control of multimorbidity (45). Effectively managing pain is essential for improving quality of life and reducing the complications associated with concurrent diseases. A healthy lifestyle and psychological well-being are recognized as important protective factors in reducing the incidence of multimorbidity. This aligns with previous studies, which show that self-health-promoting behaviors can significantly decrease the onset and progression of chronic diseases (46), particularly in the older population.

The subgroup analysis by gender indicates that age, pain, and depression influence the number of comorbidities in chronic diseases, aligning with the primary results of this study, thereby confirming the robustness of the findings. However, due to the uneven distribution after excluding missing values, some bias may have been introduced. Therefore, based on network comparisons, the study analyzed the differences in co-morbidity networks between genders. The results revealed statistically significant differences in the overall network comparisons between males and females, suggesting that gender plays a significant role in the development of multimorbidities, thereby mitigating bias associated with simple outcome-based quantitative measures. The analysis attributes these differences to significant gender disparities in dietary habits, exercise, and other lifestyle factors among middle-aged and older adult individuals in China. These differences contribute to variations in the manifestation and association of various diseases between genders (47). Furthermore, the most notable difference was in intensity centrality: male adults showed the highest value for memory-related diseases, while female adults showed the highest value for heart disease. This suggests that primary care institutions should focus on early screening and attention to cognitive impairment and degenerative diseases in middle-aged and older adult male patients, due to the limited research on gender differences in comorbidity networks, future studies should focus on this area to develop targeted disease prevention and management strategies for both male and female populations.

The methodological limitation of this study is that, although a comorbidity network was developed using data from the CHARLS (2018) with the aim of visualizing the overall morbidity status. The network only reflects the current state of chronic disease morbidity and the epidemiological trends across different demographic characteristics, without capturing deeper network differences. Future research will aim to balance covariate levels through propensity score matching and analyze the impact of different variables on the chronic disease comorbidity network. This approach will allow for a more accurate assessment of the differences in chronic disease prevention and control across subgroups. The insights gained will provide valuable guidance for public health authorities in different regions to develop tailored public health policies based on the demographic characteristics of their populations.

The limitation of the data source is that chronic disease data in CHARLS primarily relies on self-reported responses, which may introduce recall and reporting biases, potentially compromising accuracy. The cross-sectional design limits the ability to establish causal relationships, highlighting the need for longitudinal studies to validate observed associations more robustly in future research. Additionally, the analysis omitted some potentially significant factors, which may limit the comprehensive interpretation of the results. Future studies should employ longitudinal data to explore the temporal dynamics of multimorbidity. Interdisciplinary approaches integrating molecular biology and social behavior are also needed to investigate the intrinsic mechanisms of multimorbidity. These efforts could facilitate the development of multidimensional intervention strategies for high-risk middle-aged and older adult populations, ultimately improving the coverage and effectiveness of chronic disease management.

## 6 Conclusion

This study systematically analyzed multimorbidity patterns and their influencing factors in the Chinese middle-aged and older population. The data were analyzed at three levels: overall network, key influencing factors, and individual characteristics. Cardio-metabolic diseases were identified as a core component of the multimorbidity network. The findings indicate that advanced age, pain, and depression are independent risk factors affecting the number of multimorbidities. Conversely, healthy behaviors were identified as significant protective factors that reduce the number of multimorbidities. This study deepens the understanding of the mechanisms of multimorbidity and provides a scientific basis for public health interventions and policy formulation. The study highlights the importance of behavioral modification, health education, and social support, providing a scientific reference for targeted interventions for high-risk groups in public health policies.

## Data Availability

The datasets presented in this study can be found in online repositories. The names of the repository/repositories and accession number(s) can be found below: https://charls.pku.edu.cn/.
