# Supplementary material for: Network analysis of chronic disease among middle-aged and older adults in China: a nationwide survey
Source: Front Public Health. 2025 Apr 9;13:1551034. doi: 10.3389/fpubh.2025.1551034 (PMC12016668; doi:10.3389/fpubh.2025.1551034)
Supplement: Supplementary file 2 [file Table_1.DOCX]

data_female<-data%>%select(2,13:26)%>%na.omit()%>%filter(gender=="Male")%>%select(-gender,-Cancer)

data_male <- data %>%select(2,13:26)%>%na.omit()%>%filter(gender=="Female")%>%select(-gender,-Cancer)

data_female <- as.matrix(data_female)

data_male <- as.matrix(data_male)

nct_results <- NCT(

data1 = data_female,

data2 = data_male,

it = 2000, # 迭代次数

test.edges = TRUE, # 测试边的差异

test.centrality = TRUE, # 测试中心性差异

paired = FALSE, # 数据是否配对

progressbar = TRUE, # 显示进度条

centrality = c("strength", "expectedInfluence", "closeness", "betweenness") # 去掉不支持的指标

)

print(nct_results)

summary(gender_compare)

gender_compare$einv.pvals

gender_compare$diffcen.pval

gender_compare$glstrinv.sep

gender_compare$glstrinv.real

gender_compare$glstrinv.pval

gender_compare$einv.pvals

centrality_data <- data.frame(

Node = rownames(nct_results$diffcen.pval), # 提取节点名称

Strength = nct_results$diffcen.pval[, "strength"],

ExpectedInfluence = nct_results$diffcen.pval[, "expectedInfluence"],

Closeness = nct_results$diffcen.pval[, "closeness"],

Betweenness = nct_results$diffcen.pval[, "betweenness"]

)

library(reshape)

# 将数据转换为长格式

centrality_long <- melt(centrality_data, id.vars = "Node", variable.name = "Centrality", value.name = "PValue")

# 绘制条形图

if (!require("ggplot2")) install.packages("ggplot2")

library(ggplot2)

# 假设 centrality_long 是图片中的数据框

# 绘制条形图

ggplot(centrality_long, aes(x = Node, y = -log10(value), fill = variable)) +

geom_bar(stat = "identity", position = "dodge") + # 分组柱状图

geom_hline(yintercept = -log10(0.05), linetype = "dashed", color = "red") + # 显著性水平线

labs(

title = "Centrality Invariance Test",

x = "Node",

y = "-log10(P-value)",

fill = "Centrality"

) +

theme_minimal() +

theme(axis.text.x = element_text(angle = 45, hjust = 1)) # 节点名称倾斜以避免重叠
